# Supplementary material for: ‘Obstetricians’ perceptions of midwifery-led care in Bangladesh – A qualitative study
Source: PLOS Glob Public Health. 2025 Dec 12;5(12):e0005504. doi: 10.1371/journal.pgph.0005504 (PMC12700457; doi:10.1371/journal.pgph.0005504)
Supplement: S1 Text — (DOCX) [file pgph.0005504.s001.docx]

**Participant Information Sheet**

**Exploring Obstetricians's perceptions of Midwifery-Led Care in Bangladesh**

**Participant Information Leaflet**

My name is Fflur Dafis and I am studying a masters of Public Health at the Liverpool School of Tropical Medicine. I would like to invite you to take part in my research study. Before you decide, please take some time to read this participant information sheet, to understand why the research is being done and what this would involve for you. If you have any questions or if anything is unclear, please email me using the email address provided.

**Purpose of this study**

The purpose of this study is to explore the perceptions of obstetricians of midwifery-led care in Bangladesh. This study aims to document how midwifery-led care is perceived by obstetricians, who are key stakeholders in maternal care.

**Why have you been invited?**

As an obstetrician who is involved in clinical work, education, or research, you have been invited to take part in this research. No target has been set for the number of participants, but I aim to interview between 10-15 obstetricians.

**Do I have to take part?**

No, it is up to you to decide whether to join the study. Please read through this information sheet to understand the purpose of this study, if you have any questions please email me. If you agree to take part, we will then ask you to sign a consent form*.* You are free to withdraw at any time, without giving a reason.

**What will happen to me if I take part?**

If you decide to take part in this study we will contact you to arrange a suitable date and time for a face-to-face interview. I do not have a base in Bangladesh, and therefore will ask you to identify a safe, quiet location which you would be comfortable to be interviewed. I will be carrying out all of the interviews. We expect that the interview should last no more than an hour. The interviewing stage of the study will last approximately three weeks. Interviews will be audio recorded to ensure the credibility of the results and accurate transcription. Unfortunately, if you do not wish to be audio recorded, you will not be able to take part in the interviews. All recordings will be stored securely and deleted within 48 hours of the interview taking place. Any information you provide will be kept confidential and will only be included in reports or publications anonymously.

**Compensation**

We are not able to provide financial compensation for taking part in an interview, however, refreshments will be provided.

**What are the possible disadvantages and risks of taking part?**

One risk of participating in the study is that something you say could be made public. To ensure that data is stored securely, we will use an encrypted online program to store and analyse the data. The privacy of the interview location will depend on the location chosen, but we will try our best to ensure that it is a quiet area, where the interview will not be overheard. There is also a risk that although what is said in the interview will be anonymised, due to the small sample size you may be identifiable from quotes included in the study.

**What are the possible benefits of taking part?**

Taking part in this study may not directly benefit you as a participant, but we hope that the research will help gain a better understanding of how midwifery-led care is viewed, especially the potential barriers to it, which could help improve midwifery-led care and maternal care as a result.

**What will happen if I don’t want to carry on with the study?**

If you choose to participate in the study, you are free to withdraw from the study at any time during the interview process, and you do not need to provide a reason. After the information has been anonymized (within 48 hours after the interview has taken place), it will not be possible to remove your data from the analysis process.

**Will my taking part in this study be kept confidential?**

All the information about you will be handled in confidence, and stored securely on password-protected computers. Your data will be looked at by responsible and authorised personnel and representatives from regulatory authorities, who have a duty of confidentiality. Although your data will be anonymized at transcription, due to the relatively small sample size, it is possible that you may be identifiable by your quotes if you express strong views.

**What will happen to any data I give?**

After the interviews, the recordings of the interviews will be transcribed and anonymized within 48 hours of the interview, to remove any reference to your name or post with any organisation. After which the audio recordings will be deleted within 48 hours of the interview. Anonymised data may be stored for further analysis or research purposes by members of the research team only. The dissertation will be submitted in September 2023, and the anonymised transcripts will be deleted within 3 years of submission.

Your data will be handled in accordance with the [UK Data Protection Act 2018.](https://www.gov.uk/data-protection) You can find out more about how we use your information at [https://www.lstmed.ac.uk/privacy-statement.](https://www.lstmed.ac.uk/privacy-statement) LSTM Data Protection Officer can be contacted if you have any concerns about the collection or storage of your personal data: [dataprotection@lstmed.ac.uk](mailto:dataprotection@lstmed.ac.uk). The Centre for Injury Prevention and Research Bangladesh’s data protection guidelines will also be followed. If you have any complaints about the handling of your personal data, you can contact the UK Information Commissioners Office: <https://ico.org.uk/make-a-complaint/>

**What will happen to the results of the research study?**

The results of this research will be included in my dissertation for my public health masters, I hope to subsequently be able to publish it in a suitable peer reviewed journal. If you wish, we can send you a copy of the final report or publication electronically. You will not be identified in any report/ publication.

**Safeguarding**

The study team and data collectors are expected to behave ethically and responsibly at all times and follow the LSTM/Centre for Injury Prevention and Research Bangladesh code of conduct. This means that they must not ask you for any financial, physical or sexual favours in return for taking part in this research. You may also raise a safeguarding concern directly with LSTM Designated Safeguarding Officer Philippa Tubb on +44 (0)151 705 3744/safeguarding@lstmed.ac.uk. LSTM’s safeguarding commitment is described on [LSTM Safeguarding webpage.](https://www.lstmed.ac.uk/about/safeguarding)

**Complaints**

If you have a concern about any aspect of this study, you should ask to speak to the researchers who will do their best to answer your questions Fflur Dafis [email: 578201@lstmed.ac.uk]. If you remain unhappy and wish to complain formally, you can do this by contacting LSTM: [MScEthics@lstmed.ac.uk](mailto:MScEthics@lstmed.ac.uk) or CIPRB: [info@ciprb.org](mailto:info@ciprb.org), [+880258814988](tel:+880258814988).

As study Sponsor, LSTM has insurance to cover this research study, which includes compensation cover in the event that any claims arise from participation in the study.

**Sponsorship and Funding**

This project has been reviewed and been approved by LSTM Masters Review Panel and Centre for Injury Prevention and Research Bangladesh). This study does not receive any external funding.

**Contact Details**

Miss Fflur Dafis, MPH student, Liverpool School of Tropical Medicine, Pembroke Place, Liverpool, L3 5QA, UK. Tel: +44 7445673769, Email: 578201@lstmed.ac.uk

Bangladesh contacts:

Prof. Dr. MA Halim, Director, RCH dept., CIPRB email:halim.ogsb@gmail.com

Dr Abu Sayeed Abdullah – (MPH, MS, DVM) CIPRB, email: sayeedciprb@gmail.com CIPRB – number: [+880 2-58814988](https://www.google.com/search?q=ciprb+bangladesh&rlz=1C5GCEM_enGB1026GB1026&oq=CIPRB+bangladesh&aqs=chrome.0.35i39j46i175i199i512j69i64.2924j0j7&sourceid=chrome&ie=UTF-8)

LSTM contact: Terry Kana, email: terry.kana@lstmed.ac.uk, Liverpool School of Tropical Medicine, Pembroke Place, Liverpool, L3 5QA, UK.

Thank you for considering this request to participate. I am happy to answer any questions, the best way to reach me would be by the above email.

If you understand what you are being asked and are willing to participate, please read and sign the consent form below.
