# Supplementary material for: ‘Obstetricians’ perceptions of midwifery-led care in Bangladesh – A qualitative study
Source: PLOS Glob Public Health. 2025 Dec 12;5(12):e0005504. doi: 10.1371/journal.pgph.0005504 (PMC12700457; doi:10.1371/journal.pgph.0005504)
Supplement: S2 Text — (DOCX) [file pgph.0005504.s002.docx]

**Topic Guide**

**Exploring Obstetricians’ Perceptions of Midwifery-Led Care in Bangladesh**

**Introduction**

- Thank the participant for making time and travelling for the interview.
- Introduce yourself. Explain that the purpose of the interview is to explore obstetricians’ perceptions of midwifery-led care.
- Ensure each participant has a copy of the information sheet. Obtain informed consent.
- Ensure key aspects from the information sheet are well-understood, primarily:
  1. That the discussion will last a maximum of 60 minutes
  2. That the content of the interview will remain confidential
  3. That the participant’s name will not be used when reporting the findings
  4. That any quotations used will be anonymised
  5. A voice recorder will be used, only to ensure that all the information from the interview is captured, and only if they agree to being recorded.
- Explain that we are not looking for right or wrong answers but want their opinions/thoughts

**Profile of the participant**

- Post/Job title of the participant
- Department/Unit/Organisation
- Employment history
- Where did you train
- Do you work for the public/private sector/both
- Are you involved in clinical care/education/research

**Maternity service provision**

1. Could you describe how maternity care is provided in Bangladesh?

1. Who is the lead health cadre?

- What are their responsibilities?

1. What other health cadres contribute to the maternity workforce, e.g. the midwives

- What are their roles?
- Where are they working?
- Are there any restrictions on their practice?

**Professional Education**

1. In your undergraduate training, did your obstetrics secondment and education include discussion on midwifery care?

- If so – what was said/how was it portrayed

1. During your speciality training, was midwifery practice discussed

- In global context, or in Bangladesh context

**Professional experience**

1. With the introduction of the midwifery cadre, what are your experiences of (positive and negative):

- Policy discussions
- Regulation
- Advising the curriculum
- Teaching and mentoring
- The work environment

1. From your experiences, what do you think of midwifery-led care?

- Examples of positive experiences
  - - Examples of negative experiences
    - Has this affected your workload

1. With the rollout of midwifery-led birth units, what do you think is the future of midwifery-led care within the maternity services in Bangladesh?
   - - What are the enablers of this
     - What are the barriers to this
     - What would you recommend for this

1. Do you envisage a role for yourself in facilitating or supporting the rollout of midwifery-led care?

- If yes – what is it?
- If not – for what reason?

**Additional information**

Do you have a colleague you recommend that I speak to as part of this study?

**Closure**

- Is there anything else you would like to add?
- Summarise the main points made, and check that the participant agrees
- Thank participant for their time
