# Supplementary material for: ‘Obstetricians’ perceptions of midwifery-led care in Bangladesh – A qualitative study
Source: PLOS Glob Public Health. 2025 Dec 12;5(12):e0005504. doi: 10.1371/journal.pgph.0005504 (PMC12700457; doi:10.1371/journal.pgph.0005504)
Supplement: S1 Table — (DOCX) [file pgph.0005504.s003.docx]

|  | **Support and monitor in uncomplicated pregnancies** | **Grouped together with nurses** | |
| --- | --- | --- | --- |
| **Diverse understanding of midwives’ scope of practice** | *“They can [conduct] delivery, normal cases, easy, normal delivery. But when they face complication, that is prolonged labour, obstetric labour, malpresentation, they refer.”*   - *Consultant, female, private experience only* | *“In our hospital we cannot differentiate between nurse and midwife.”*   - *Registrar, female, private experience only* | |
|  | **Improving access and equity** | **Decreased workload** | **Specialised practitioners** |
| **Perceived benefits of midwifery** | *“…doctors are not available at the grassroot level...So if we can establish increased number of [midwives], they can handle the situation. Patient need not refer. There may be riverside people, peripheral people. There is no communication, road is not available. So they can serve the people there, at least primary care. Normal delivery. So it is very important.”*   - *Consultant, female, private experience only*   *“Suppose you [have] three patients, I allocate 5 minutes, 5 minutes, 5 minutes. She needs 1 minute, you need no minute…she needs half an hour. Now if I allocate everyone 5 minutes, I am doing an injustice to her...So this is why, are we doing too much or too less. So distribution of the risk involved matching with the scenario, that is very important.”*   - *Professor, male, public and private experience*   *“People like us country like us, where our limited resource. But we want to maximise this resource by involving the midwife”.*   - *Professor, male, public and private experience* | *“…doctors are very busy. Normal delivery is a long procedure and takes a long time. If we want to say that we increase normal delivery rate, safe delivery rate, and we want supervision of our mothers with respectful maternity care…if we engage particular groups like midwives…it’s possible to take care of our safe deliveries.”*   - *Consultant, female, private experience only*   *“In our hospital there is midwife…she is very skilled, she conducted the delivery, she can inform us about the progress of the labour very efficiently, so we can rely on her, we can see the other patients here, in labour room she is monitoring the patient and informing us over telephone. If she faces any difficulties, she can inform us…she is very skilled. This changes our job. Because we can give more time here.”*   - *Consultant, female, public and private experience* | *“Rural area, midwives delivery individually. By practice they are skilled.”*   - *Senior consultant, female, public and private experience*   *“…they are very much dedicated…that is because they work only in narrow sector, they are very much well [knowledgeable] about this”*   - *Consultant, female, public and private experience*   *“They’re good at learning about normal delivery and other things. They are so much*  *friendly. And they have a knowledge about how to care for the mothers.”*   - *Consultant, female, private experience only* |
|  | **Integration and acceptance of midwives into the healthcare system** | **Lack of trust in their competency** | **Lack of trust in their education** |
| **Factors restricting midwives’ professional autonomy** | *“I think there will be some problems arising when both of them come…If you think that doctor cannot do a proper delivery, and that midwife is good at delivery, then there will be a problem. If it is happening that senior is liking that midwife but she is not liking that doctor…this type of problem will arise”*   - *Assistant professor, female, private experience only*   *“Previously sisters was a third class citizen, and nowadays they are second class citizen. And we are 1st class citizen… But I think…they must come for Bangladesh.”*   - *Assistant professor, female, private experience only* | *“They cannot understand when there is prolonged labour, malpresentation, malposition…They call for us late…There sometimes is mishap, where patient needs NICU submission, emergency c-section, but party is not ready for caesarean section, they know that everything is okay, then what happens now, in the eleventh hour? They cannot understand all cases…”*   - *Consultant, female, private experience only*   *“…in any difficult situation they refer. But in such a critical condition that patients come. Sometimes that patient lose their life in ambulance.”*   - *Senior consultant, female, public and private experience*   *“Even vulval haematoma they cannot identify”*   - - *Senior consultant, female, public and private experience* | *“But in our country, they are not posted during their training period, so how can they train to delivery individually [deliver on their own]? They only learn but not do. They not do in practical scenario, so they are not efficient.”*   - *Senior consultant, female, public and private experience* |
|  | **Government promotion** | **Curriculum adaptation** | **Increased clinical experience** |
| **Strengthening future midwifery** | *“So there should be more work from the government level. For publicity about their work..”*   - *Consultant, female, public and private experience* | *“…they should improve curriculum about knowledge, and also practical. They have to … perform more deliveries so they have experience, that complications like breech delivery, normal delivery prolonged, partograph showing there may be some problem so I need to inform or call doctor earlier.”*   - *Consultant, female, private experience only* | *“Midwifery course. That should be dramatic change. Because obstetricians should teach them. Not nurses. Because that is not possible, obstetrician should teach…1 or 2 or 3 months is not enough. At least 6 months to one year training in labour room, is very much important.”*   - *Senior consultant, female, public and private experience*   *“Proper training, ensure her working area is safe, that is very important. Her working atmosphere should be safe…Working atmosphere, she should feel that she is comfortable at discharging her duties.”*   - - *Professor, male, public and private experience* |
