# Supplementary material for: ‘Obstetricians’ perceptions of midwifery-led care in Bangladesh – A qualitative study
Source: PLOS Glob Public Health. 2025 Dec 12;5(12):e0005504. doi: 10.1371/journal.pgph.0005504 (PMC12700457; doi:10.1371/journal.pgph.0005504)
Supplement: Attachment — Submitted filename: letter to reviewers.docx [file pgph.0005504.s006.docx]

Thursday 29^th^ of May,

Dear reviewers,

Thank you very much for your feedback. Please find below my response to your comments.

**Reviewer #1 comments:**

- Introduction lines 42-43: it is a bold statement without any supporting reference. I would suggest adding a reference or rephrasing the opening line.
  - I changed ‘unfamiliar’ to ‘relatively new’ referencing this.
- The introduction has been well constructed, but could be organised better, so that the text flows from current situation, context, facilitators, barriers and their impact on the profession, ending with why obstetricians' perceptions and experiences mattered and formed the core for this piece of work. Right now a lot of back-and forth happens in the text which may be confusing to readers outside of the region.
  - I have rearranged the introduction to improve the flow.
- Methodology: lines 82-84: I wonder whether data saturation was possible with just 13 "semi-structured" interviews. Do the authors mean in-depth interviews? If not, a paragraph is needed on how was data saturation identified and consented upon by all co-authors.
  - I have amended this to ‘in depth’.
- line 121 Table 2: Consenting to or not consenting to participate cannot be called an inclusion or exclusion criteria. If they did not consent, it implies they were eligible for inclusion - thus must have met inclusion criteria - but refused participation. Therefore, I am requesting deletion of this criterion from the table.
  - I have amended the table.
- lines 124-132: interviews that lasted barely 10 min raise significant concerns in my opinion, in a qualitative study. how many interviews ended in less than 40 min? Even 40 min interviews could be considered just adequate in terms of allowing an in-depth/holistic exploration of issues discussed, especially looking at the thematic guide (Appendix A). Authors need to justify conducting interviews in English/translation into English during the interview itself, rather than employing local interviewers to conduct full interviews in Bangla, especially in the light that authors knew the obstetricians to be busy and, then taking the dual language interviews that would have further cut into the actual discussion time of 10-40 min interviews.
  - The majority of interviews lasted between 30-40 minutes. With shorter interviews generating recurring themes to the longer interviews.
  - Obstetricians receive their medical training in English, were fluent and happy to be interviewed in English. Translation was only necessary for certain words/phrases that interviewees were unsure of.
- Lines 174-178: It will be great if you could add the approval certificate numbers from both the institutions.
  - I have been unable to locate the approval certificate numbers for the ethical approval as these were not on the certificates I received.
- Lines 184-188: Authors state that the recordings were deleted within 48 hours, after the transcription was complete. I would like to better understand how was the analysis completed without the recordings, considering the pressure put on certain words, tone, and associated voice inflections are all qualitative data, especially in emotive coding which is commonly used for perceptions and experiences studies. Sometimes the weight put by the participants on certain words changes the intention drastically (from serious reflection to a taunt or a patronizing remark for example). If the authors captured all the inflections via detailed transcription, this should be made clear.
  - I have provided more detail on the depth of the transcription to capture these nuances.
- Overall, I request the authors to reflect on the interchangeable terms that inadvertently mix the midwife-led care philosophy with the medical model of care philosophy. For example: reflect on your positionality on birth/childbirth vs. delivery; women/mothers vs. patients and so on.
  - I have amended these throughout the paper to births/mothers.
- The manuscripts presents some very interesting findings, but for the most part, the quotes are too short and too few to establish that the findings are rooted in actual interview data. Consider giving longer quotes or several short ones to demonstrate how you reached to the conclusions that you reached, with all the rich context outlined in your text.
  - I have extended the length of existing quotes and added a few additional.
- Lines 436-438: authors mention family planning services. Sorry if I missed it, but I did not see any reference to FP in the results section.
  - I have amended this.
- Limitations: I feel the authors should add the study strengths as well, since this is a critical evaluation of the research process which may help future researchers exploring midwifery research in Bangladesh. Kindly look at my comments to further elaborate in this sections.
  - I have moved a section from the methodology section to the strengths an limitations section, discussing the strengths of qualitative research methods.
- Recommendations: Could be bolder, in light of your strong findings.
  - I have expanded on my recommendations.

**Reviewer #2 comments**

- Title: A suggestion is: Obstetricians’ perceptions of midwifery led care in Bangladesh – A qualitative study
  - Thank you for your suggestion, I have amended the title.
- Line 22: Is it midwifery that is a relatively new profession or is it the midwife as a separate profession?
  - I have reworded this sentence to say “The midwife as a separate and autonomous profession is a relatively new concept in Bangladesh, as a result, its integration into the maternal healthcare system is yet to be determined”
- Line 22-23: To better link the sentence to Obstetricians (line 23), the first sentence needs to include …. integration into the maternal healthcare system
  - I have amended this.
- Line 1: Suggest changing this reference to: The historical development of the midwifery profession in Bangladesh 2017 (Bogren, Begum, Erlandsson)
  - Thank you for the suggestion, I have changed this reference.
- Line 66: The sentence states that the introduction of midwifery in Bangladesh has been well documented. This is true and needs to be supported by a reference.
  - I have added references to support this.
- To understand your findings, your introduction would benefit from a few sentences about how to become a midwife in Bangladesh. Several scientific papers have discussed this and proven that this education aligns with the ICM competencies for midwives.
  - I have added information on how to become a midwife in Bangladesh – “To become a midwife in Bangladesh individuals must study a three year diploma course, the curriculum of which aligns with the ICM competencies (8, 10). 61% of the institutions required students to be able to write in English, and 32% required them to be able to write in Bangla (11){Bogren, 2021 #72}. Aspiring midwives are also required to achieve a Higher Secondary School Certificate (12). However, according to a study by Bogren et al., only 34% of the nursing institutes which provide midwifery education had a transparent admissions policy, therefore it is unclear of the criteria required by prospective midwifery students (11)”.
- Line 78: The table of aims and objectives does not benefit the paper. The objectives could be presented as your research questions. Objective 2 is similar to the aim and can be removed.
  - I have amended this table to be research questions.
- Line 82-84: Please add a reference after a qualitative study.
  - I have added a relevant reference.
- Line 86- 97: This paragraph would suit better in the strengths and limitation section.
  - I have moved this paragraph to the strengths and limitations section.
- Please add a heading Setting, which will help the reader to understand in what context the study was taken place. Ex Number of births (Vaginal/CS), Number of obstetricians, midwives, nurses etc.
  - I have added a subheading ‘setting’ to provide some context.

“Bangladesh, with a population of 169,356,251, is the 8th most populated country in the world (17). Since 1980 there has been an impressive decline in poverty, from 41.9% to 13.5% in 2016 (18). In 2015, they attained lower-middle-income status, and by 2031, aim to reach upper- middle income status (18).

According to the latest DHS survey, the caesarean section rate of live births in Bangladesh was 45% (19). This is compared to the estimated global average of around 21% (20).

The DHS also states that 56% of births were assisted by a qualified doctor, 19% by untrained traditional birth attendants, and 13% by a nurse, midwife or paramedic (19). These statistics demonstrate a clear lack of midwifery specific data.

A report by WHO states that there is an estimated 67 midwives per 10,000 population, and 317 nurses per 10,000 population. Of these 67 midwives per 10,000, 63 worked in the urban setting, and only 4 rurally. It also states that there are 635 medical doctors per 10,000 but does not state the number of obstetricians specifically. (21)”

- Line 118: Please elaborate about how the participants were recruited (face-to face, email, advertisement etc).
  - I have now explained that participants were recruited face to face.
- Line 121: Reconsider how your inclusion and exclusion criteria are presented, as it is presented now, your exclusion criteria is just the opposite of your inclusion criteria, which is redundant.
  - I have amended this table.
- Line 127: Please give some examples of your key questions to give the reader an understanding about what questions that were asked (even though your interview guide is attached)
  - I have provided examples of questions asked during the interviews.
- Line 134: I am a bit confused about the key informant. A Key informant is a person being interviewed and should therefore be part of the data collection. If I understand in correct, you present this person’s opinion in the discussion, when you discuss your results. See Example line 513. Why not using scientific articles to contextualize and address your results? What says that this person knows better than your participants and published scientific papers. Out of curiosity, was this person a midwife? Because that could give some nuanced tone to the discussion. However, there are so many publications out there made by experienced midwives with extensive knowledge of midwifery in Bangladesh.
  - I have removed the key informant from the discussion section of the paper, and instead used a reference.
- Line 150-157: The thematic analysis needs to be further clarified. How were the codes identified, how did you identify the categories, vs themes
  - I have provided more detail on the thematic analysis.

“Using NVivo software, transcriptions were coded to fragment the data, based on specific thoughts and ideas. Related codes were grouped together to form subthemes. The relationships between these subthemes were further analysed to determine how they addressed the research questions, leading to the formation of broader themes. The coding process was guided by the data itself and by our research questions. Thematic analysis is subject to the researchers’ judgement, to mitigate this risk of bias, themes were discussed with supervisors in Bangladesh and in Liverpool, as well as with the research assistant. These discussions helped refine and finalise the themes. The naturalistic paradigm was core to this research. Perceptions are subjective, therefore understanding their complexity and context is essential to fulfil the research aim (24).”

- What reference did you use for your thematic analysis? You mentioned that you made categories, usually in thematic analysis, themes and sub themes are used.
  - I have amended this to themes and sub themes rather than categories.
- To strengthen your analysis please provide an analysis matrix to exemplify the process.
  - I have added my analysis matrix as an appendix.
- Please also include a table presenting themes and respective sub themes to provide an overview of your results.
  - I have added this table in text.
- Please use the term birth and childbirth instead of delivery. Women give birth.
  - I have now changed this throughout the paper. However the term ‘delivery’ does appear often in the quotes.
- Line 347: Please consider changing the theme to Lack of trust in midwives’ clinical education
  - I have changed this theme.
- Line 443-445: Starting with the sentence a Key informant…..This information does not add to a scientific discussion. Suggest deleting,
  - I have deleted this from the discussion.
- Line 513: I would suggest referring to a scientific paper Instead of referring to Unfpa staff. There are several of published articles about midwifery education and educators in Bangladesh.
  - I have referenced a systematic review.
- Do you have any suggestions on how to close the disconnect between obstetricians’ understanding of the role and scope of practice of Bangladeshi midwives and international definitions? Because, reading your findings made me quite sad after so many years of investments in building a midwife profession in Bangladesh.
  - I have expanded on my recommendations.

Yours sincerely,

Fflur Dafis
