# Supplementary material for: ‘Obstetricians’ perceptions of midwifery-led care in Bangladesh – A qualitative study
Source: PLOS Glob Public Health. 2025 Dec 12;5(12):e0005504. doi: 10.1371/journal.pgph.0005504 (PMC12700457; doi:10.1371/journal.pgph.0005504)
Supplement: Attachment — Submitted filename: letter to reviewers 2 .docx [file pgph.0005504.s007.docx]

22^nd^ of September 2025

Dear reviewers,

Thank you very much for your feedback, and I am very sorry for my delayed response.

Please find below my response to your comments.

1. There are several grammatical errors, such as inappropriate use of commas and apostrophes. There is a mix of present and past tenses in your methods and findings sections, both are traditionally written in past tense. This could be corrected by getting the manuscript reviewed by an English language expert.

I have reviewed the grammar and punctuation throughout this.

2. What is CIPRB (line 131)? Requesting you to include full forms of all acronyms at the time of first use.

This is specified on line 127.

3. Authors describe trustworthiness improving measures in detail (Lines 184-196). How about the other three measures: confirmability, credibility and transferability?

I have now detailed specifically how each of these measures contribute to the overall trustworthiness of the study, as well as dependability the fourth pillar of trustworthiness.

4. Reviewer #2: Thank you for addressing my feedback. I appreciate that you have removed the comments from the Key informants from the Discussion. Please reconsider if you need the section about the same in the method section. I would suggest to remove it, as this is not presented in the analysis.

Thank you for your comment, I have removed this section.

Yours sincerely,

Fflur Dafis
